# Incidental Detection of Neuroendocrine Carcinoma of Rectum During Staging Workup of Renal Cell Carcinoma

**DOI:** 10.14740/wjon949w

**Published:** 2015-12-31

**Authors:** Kumaresh Athiyappan, Rajoo Ramachandran, Swaminathan Rajendiran, Vinoth Thangam

**Affiliations:** a Department of Radiology and Imaging Sciences, Sri Ramachandra University, Porur, Chennai, Tamilnadu, India; b Department of Pathology, Sri Ramachandra University, Porur, Chennai, Tamilnadu, India

**Keywords:** Neuroendocrine tumor, Renal cell carcinoma and carcinoid

## Abstract

Malignancies of rectum and kidneys are common pathologies in clinical practice; however, the incidence of these malignancies coexisting together is unclear. The main purpose of this article was to show the usefulness of computed tomography (CT) in diagnosing these rare synchronous tumors. We report a case of neuroendocrine carcinoma of the rectum in a 57-year-old male patient who came for staging workup of renal cell carcinoma (RCC) of the left kidney. To our knowledge, this is the first case of synchronous RCC and rectal neuroendocrine carcinoma coexisting in the same patient.

## Introduction

Neuroendocrine tumors of the rectum account for approximately 19% of gastrointestinal neuroendocrine tumors (NETs) [1]. The vast majority of tumors are asymptomatic and detected incidentally during colonoscopy or endoscopy. In general, NETs arise from the amine precursor uptake and decarboxylation cells. These tumors are most commonly found in the gastrointestinal tract and are located in decreasing order of frequency in the ileum, rectum, appendix, stomach, duodenum and jejunum and colon [2]. In this case report, we present our experience in a patient who came for diagnostic workup of renal cell carcinoma (RCC) and a rectal mass was detected incidentally during CT imaging which turned out to be a neuroendocrine carcinoma on histopathologic examination.

## Case Report

**Figure 1:**
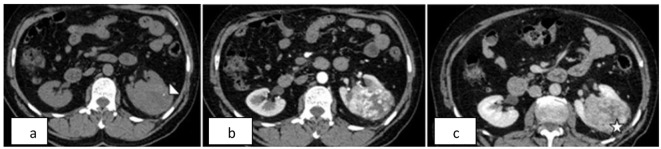
Left renal mass. Non-contrast and contrast-enhanced axial CT of the abdomen shows a well-defined mass in the interpolar region of the left kidney. a) Non-contrast CT shows a small focus of calcification (arrow head) within the mass. b) Arterial phase image shows heterogenous and intense enhancement. c) Venous phase image shows relative washout with areas of necrosis (asterisk) within the mass.

**Figure 2:**
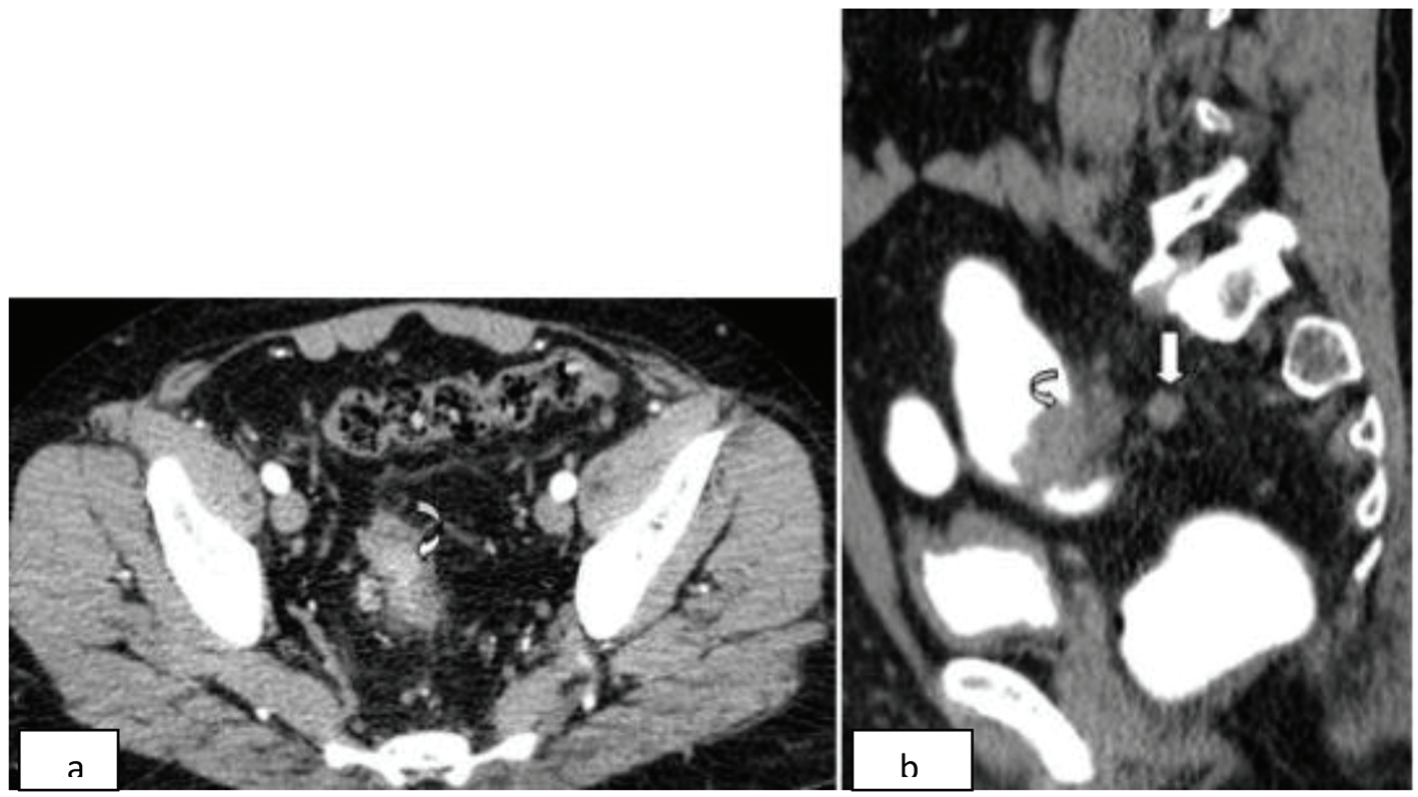
Rectosigmoid mass. a) Contrast-enhanced axial CT in arterial phase shows a well-defined moderately enhancing mass involving the rectosigmoid region (curved arrow). b) Sagittal reformatted CT after rectal contrast shows the polypoidal mass (curved arrow) infiltrating the perirectal fat with an adjacent perirectal lymphadenopathy (straight arrow).

**Figure 3:**
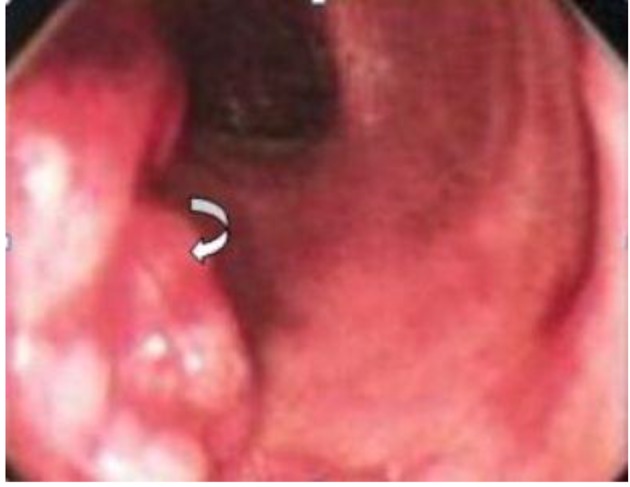
Colonoscopy shows an intraluminal polypoidal mass lesion (curved arrow) involving the rectosigmoid region.

**Figure 4:**
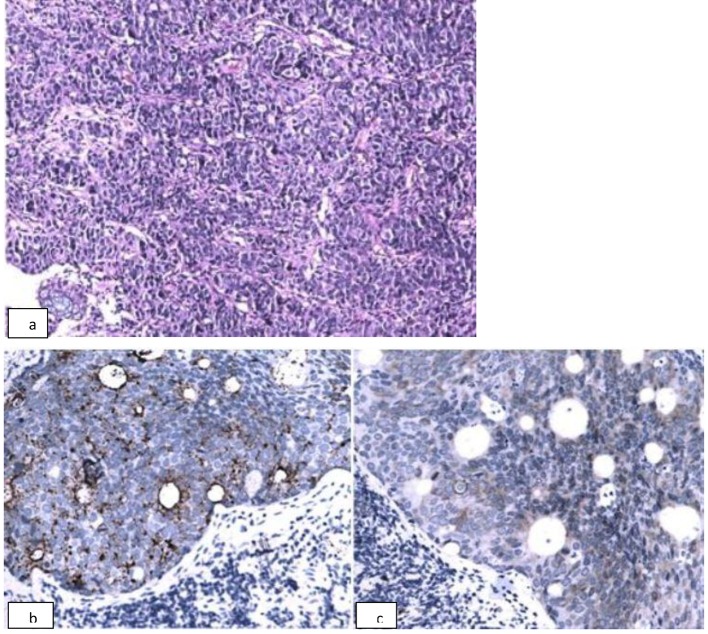
Histological confirmation of the diagnosis of poorly differentiated neuroendocrine carcinoma of rectum. a) Photomicrograph of hematoxylin and eosin stained biopsy sample of the rectosigmoid mass shows small round blue cells arranged in zellballen pattern with nuclear molding. Vesicular nucleus with salt and pepper chromatin is also seen (× 200). b) Immunohistochemistry of the rectal mass shows tumor cells focally positive for synaptophysin, and c) chromogranin (× 200).

**Figure 5:**
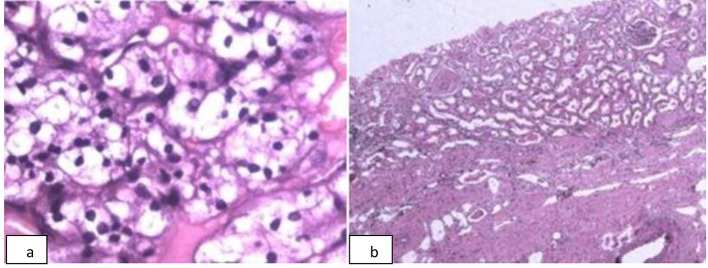
Histological confirmation of the diagnosis of renal cell carcinoma: clear cell type of the left kidney. a) Photomicrograph of hematoxylin and eosin stained section of the renal mass shows nests of clear cells with intervening thin fibrovascular septae (× 400). b) Photomicrograph of hematoxylin and eosin stained section of adjacent compressed normal kidney (× 100).

A 57-year-old male came with complaints of left loin pain and hematuria for the past 5 days, without other significant history. On physical examination, a mass was palpable on the left lumbar region. Ultrasonography (USG) of the abdomen showed a solid mass lesion with internal vascularity seen involving the left kidney. Contrast-enhanced computed tomography (CECT) of abdomen was done for further evaluation. CECT of the abdomen showed a well-defined heterogeneous soft tissue mass lesion with a tiny speck of calcification involving the interpolar region of the left kidney. The lesion was seen extending into the perinephric space and abutting the perinephric fascia seen. No extension beyond the fascia was seen. No extension into the main renal vein was seen. No lymphadenopathy was seen. The lesion showed hypervascularity in arterial phase with relative washout in venous phase images (Fig. 1a, b, c). In addition to the renal mass, there was a well-defined homogenously and moderately enhancing polypoidal intraluminal mass lesion measuring about 2.5 cm seen involving the rectum about 12 cm from the anal verge (Fig. 2a, b). Significant wall thickening and perilesional lymphnodes were noted with the largest lymphnode measuring 10 mm in short axis (Fig. 2a, b). Based on the radiological findings, a possibility of synchronous malignancy of the left kidney and the rectum was raised. The second possibility raised was a renal cell carcinoma (RCC) of the left kidney with metastasis to the rectum. Based on the radiological diagnosis, the patient was subjected to colonoscopy-guided biopsy of the intraluminal mass lesion of the rectum (Fig. 3) and sent for histopathological analysis. Histopathology showed features of poorly differentiated neuroendocrine carcinoma (Fig. 4a) and immunohistochemistry showed tumor cells focally positive for synaptophysin and chromogranin which confirmed neuroendocrine carcinoma (Fig. 4b, c). The patient underwent radical nephrectomy of the left renal mass and histopathology confirmed a grade II clear cell RCC (Fig. 5a, b). Surgical resection of the rectal mass was not done as the patient was unwilling for further surgery. The patient is started on cisplatin and etoposide chemotherapy for the neuroendocrine carcinoma and is followed up every 3 months.

## Discussion

Neuroendocrine cells are the endocrine cells that are derived from the neural crest and neural ectoderm. These cells are widely distributed in the body involving the GIT, biliary tract, lung, liver, skin and the urethra. NETs are seen commonly arising from the GIT and the pancreas [3]. In the GIT, ileum is the common site (about 30%), followed by rectum (about 21-27%), appendix (about 17-20%), stomach (6-9%), duodenum and jejunum (2-3%) and rarely in colon [2]. They produce a variety of hormones like serotonin, histamine, prostaglandins and tachykinins. Carcinoid syndrome occurs when the vasoactive amines are released into the systemic circulation. These vasoactive substances are metabolized by the liver and the syndrome is usually seen in patients with hepatic metastasis and in extraintestinal carcinoid. The syndrome presents with bronchospasm, abdominal pain, diarrhea, flushing, niacin deficiency and right-sided cardiac valvular fibrosis. The symptoms are precipitated by exertion, stress and food products like cheese and chocolates [4].

NETs are classified based on the tumor differentiation into well-differentiated endocrine tumor, well-differentiated endocrine carcinoma (malignant carcinoid) and poorly differentiated endocrine carcinoma. The well-differentiated gastroenteric NETs also called as carcinoids are generally small in size whereas endocrine carcinomas are generally ulcerative tumors with metastasis, mimicking adenocarcinoma [3]. Syndromes associated with NET are multiple endocrine neoplasia type I, neurofibromatosis type I and von Hippel Lindau syndrome.

Small bowel NETs are commonly seen in the ileum and the patients are usually asymptomatic. The symptoms can be due to the primary lesion or metastasis to the mesentery, lymph nodes and the liver. The primary lesion can cause intestinal obstruction, bleeding, perforation, intussusception and ischemia [4]. The lesion is usually small and appears as a nodular or plaque like hyper-enhancing lesion. The detection rate using conventional imaging techniques is very low whilst CT/MRI enterography/enteroclysis increases the detection rate of the primary tumor [4].

Mesenteric metastasis from an NET appears as a spiculated mass with surrounding fixity, kinking and tethering of the small bowel. This is due to the desmoplastic reaction caused by the vasoactive amines secreted by the carcinoid [5]. Scattered calcification can be seen within the mass. Fibrosis with narrowing of the mesenteric vessels can occur. There may be associated bowel wall thickening, due to submucosal fibrosis, direct infiltration of the tumor or bowel ischemia. The imaging differential diagnosis for a mesenteric metastasis from NET is sclerosing mesenteritis [6] and biopsy is needed to differentiate them. Hypervascular metastasis to liver occurs in NET.

The rectum is the second most common site for NET in the GIT next to the small bowel [3]. Rectal NETs are usually asymptomatic and are detected incidentally during colonoscopy or CT scan of the abdomen. They appear as small, solitary or multiple nodules or as a large polypoidal ulcerating mass as seen in neuroendocrine carcinoma [4] mimicking as adenocarcinoma of the colon. Carcinoids involving the rectum rarely metastasis and the metastasis are not associated with carcinoid syndrome [7] and hence rectal carcinoids have a better prognosis. Transrectal ultrasonography can be used to find the depth of invasion by the lesion.

Indium 111-pentetreotide is used in localization and staging of the carcinoid. It is also used to assess the receptor status of the disease which can predict the response to octreotide treatment [8]. Fluorodeoxyglucose-PET/CT has a limited role in well-differentiated tumor. But PET scan using gallium-68-labeled with octreotide analogs shows uptake by the tumor cells.

NETs can be multiple and can be associated with another primary malignancy. This may be due to common endogenous or an exogenous factor causing the neoplasm. Malignancies associated with small bowel NET include prostate (26%), breast (14%), colon (9%), lung or bronchus (6%) and urinary bladder (5%). So far only one case of bowel carcinoid associated with adenocarcinoma of kidney has been reported previously [9]. However, poorly differentiated NET with associated renal malignancy has not been reported in English literature. Similarly malignancies associated with RCC can be breast, prostate, colorectal and bladder cancer as well as non-Hodgkin’s lymphoma [10]. In case of NET involving the GIT or an RCC, careful evaluation is necessary to look for a second or synchronous malignancy.

Localized gastrointestinal NETs are surgically removed and in patients with liver metastasis various treatment options like surgical resection, chemoembolisation, radiofrequency ablation, long acting somatostatin analogs, chemotherapy, radionuclide therapy with ^111^In-, ^90^Y-, or ^177^Lu-labeled octreotide analogs are used [11].

### Conclusion

Carcinoid tumors are a rare group of neoplasms and considerable progress has been made in understanding the nature of these tumors. Our understanding remains poor in the coexistence of the tumor with RCC. Rectal neuroendocrine carcinomas can mimic like adenocarcinoma on imaging. Although standard surgical techniques may still provide a chance of cure, it must be carefully weighed for each individual patient.
